# Application of Ultrasound Phase-Shift Analysis to Authenticate Wooden Panel Paintings

**DOI:** 10.3390/s140507992

**Published:** 2014-05-05

**Authors:** José M. Bravo, Juan V. Sánchez-Pérez, Marcelino Ferri, Javier Redondo, Rubén Picó

**Affiliations:** 1 Departamento de Física Aplicada, Universidad Politécnica de Valencia, Av. de los Naranjos s/n, Valencia 46022, Spain; E-Mails: jusanc@fis.upv.es (J.V.S.-P.); mferri@upv.es (M.F.); fredondo@upv.es (J.R.); rpico@upv.es (R.P.); 2 Centro de Tecnologías Físicas: Acústica, Materiales y Astrofísica, Universidad Politécnica de Valencia, Av. de los Naranjos s/n, Valencia 46022, Spain; 3 Instituto de Investigación para la Gestión Integrada de zonas Costeras, Universidad Politécnica de Valencia, Paranimf 1, 46730 Grao de Gandia, Valencia, Spain

**Keywords:** ultrasound scan, authentication artwork, non destructive testing (NDT), phase-shift analysis

## Abstract

Artworks are a valuable part of the World's cultural and historical heritage. Conservation and authentication of authorship are important aspects to consider in the protection of cultural patrimony. In this paper we present a novel application of a well-known method based on the phase-shift analysis of an ultrasonic signal, providing an integrated encoding system that enables authentication of the authorship of wooden panel paintings. The method has been evaluated in comparison with optical analysis and shows promising results. The proposed method provides an integrated fingerprint of the artwork, and could be used to enrich the cataloging and protection of artworks. Other advantages that make particularly attractive the proposed technique are its robustness and the use of low-cost sensors.

## 1. Introduction

In recent years there has been growing interest in the preservation of cultural heritage [1,2]. Nowadays, the value of the artworks goes beyond their pure economic value, becoming part of the World's cultural patrimony and teaching us about the history, beliefs or state of technological development in the period when the artworks were made. It is therefore worth keeping them in good condition, continuously evaluating their state in order to preserve them from damage due to external agents like temperature, humidity or pollutants. On the other hand, the validation of the authenticity of artworks is yet another aspect to consider in culture heritage protection. To achieve these two goals several scientific diagnostic and authentication methods are used; most of them developed from applications in physical science and engineering [3]. Among them, a set of techniques generally called non-destructive testing (NDT) is particularly interesting because it allows analysis without damaging the artwork, avoiding direct contact between the artwork under study and the instruments used in the analysis.

Several NDT techniques have been developed throughout the years for the diagnosis and authentication of artworks. Thus, internal defects can be detected using infrared thermography [4], but its effectiveness depends heavily on both the diffusivity and conductivity of the analyzed sample. Other techniques used to check the internal structure and the existence of previous repairs or defects in artworks are X-ray or holographic interferometry techniques [5]. Another method is based on the correlation between the chromatics of paintings and the composition of pictorial materials using spectral and chromatic analysis [6]. Some authors have used laser Doppler vibrometry with acoustic excitation [7] or a technique based on the acoustic excitation-acquisition response to check antique frescoes [8]. Analysis of the pressure field generated by a commercial parametric loudspeaker in both the near and far fields is applied to reveal pictorial film detachments in renaissance panels [9]. Among all NDT methods used to diagnose and authenticate artworks, the non-contact ultrasonic techniques present promising results [10]. Traditionally, these techniques have been used to detect surface anomalies in structures by *in situ* relative measurement of acoustic energy absorption [11], or in the field of material characterization to identify defects or to determine the elastic properties by analyzing the behavior of the Lamb and Rayleigh waves transmitted throughout the materials [12]. In this sense, some researchers have used air-coupled acoustical imaging techniques to diagnose wooden panel paintings [13] by exciting vibrational modes in the artwork or to authenticate Goya's artwork using Terahertz waves [14]. Finally, some studies present promising results by combining NDT techniques in structural studies concerning heritage conservation and authentication [15]. As an example, the state of the 13th century rose window of Troia Cathedral has been evaluated using ground-penetrating radar, infrared thermography and ultrasonic tests [16]. Diagnosis of the inside of artworks using air-coupled ultrasounds is normally based on the analysis of elastic waves (Lamb or Rayleigh) reemitted to air, although some of us have used phase-shift analysis (PSA) to locate brick pieces under a renaissance fresco [17], demonstrating experimentally that part of the ultrasound signal penetrates the materials, and is then reflected due to changes in impedance inside the artwork.

The goal of this work is to demonstrate that the PSA technique is a robust and useful method for authenticating wooden panel paintings. The ultrasound phase obtained at each point of the artwork gives information not only on the scattering of the incident signal but also on the inner layers of paint and base carrier [17], providing an coding image of artwork.

To check the validity of our results we performed a detailed topography of the chosen paint with a scanning technique based on photogrammetric correlation. Topographic images obtained have a precision of 0.2 mm [18].

The wooden panel painting authenticated in this paper is a work by the Spanish painter José Peris Aragó belonging to a private collection, and ceded by the owner for this study. José Peris Aragó, born in Alboraya, was a painter, illustrator and poster artist who lived in the twentieth century. He was a famed landscape artist whose style sits halfway between realism and impressionism. Indeed, the impressionistic character of his work was fundamental in choosing the panel painting because it has reliefs that can be tested accurately analyzing the phase-shift with our technique.

The paper is organized as follows: In the second section we explain the theoretical foundations. Both the experimental setup and the methodology followed in this work are presented in the third section. The results are analyzed and discussed in the fourth section. The last section contains the concluding remarks, where the results are summarized.

## 2. Theoretical Foundations

Echo-impulse techniques are widely used in industry and medicine to visualize the inside of objects or the human body as a good alternative to avoid high energy radiation damage produced by other dangerous techniques based on X-ray. Highly damped transducers must be used with these techniques in order to emit and detect pulses by the time of flight. In contrast, with the PSA method used here, the setup works under steady state and low cost transducers with low damping.

**Figure 1.**
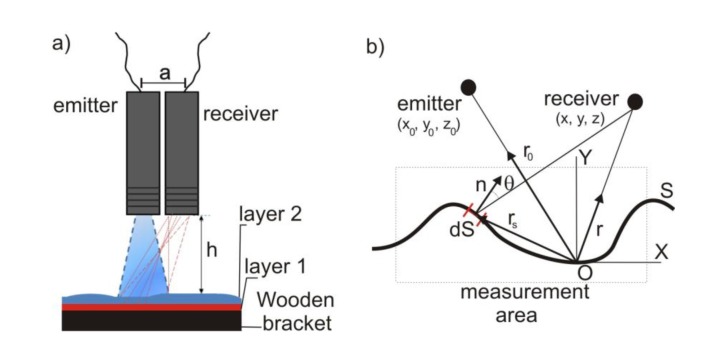
(**a**) Diagram of the phase-shift analysis (PSA) method used; (**b**) Scheme used to explain the obtaining of scattered pressure on a corrugated surface by using the Fresnel formula.

In the PSA method an ultrasonic pure tone is emitted by a transducer and impinges on the rough surface under study. A receiver located at the same height as the emitter collects the acoustic energy that interacts with the artwork (Figure 1a). Due to the roughness on the surface of the paint, ultrasound incident wave scattering is random. If we consider the variations in the elastic inside properties of the artwork due to the existence of different paint layers and/or the status of the wooden bracket, a part of the transmitted signal should be reflected. Thus, the ultrasonic signal reaching the receiver contains more information than a mere topographical description. Thus, analysis of the phase shift produced in this process provides an ultrasonic image of the pictorial work on wood as a whole.

The scattering of an ultrasonic signal impinging on a rough surface has been analyzed in the case of near field conditions by Fresnel diffraction [19]. This analytical approach provides good results to obtain the scattered complex pressure *p*(*r*) produced on a non-absorbing (with reflection coefficient *R* = 1) rough surface *S* and the emitter and receiver are considers as points:

(1) $$p(r)=-2jk\iint_{S}\frac{e^{-j\vec{k}\left({\vec{r}}_{s}-{\vec{r}}_{0}\right)}}{4\pi \left|{\vec{r}}_{s}-{\vec{r}}_{0}\right|}\frac{e^{-j\vec{k}\left(\vec{r}-{\vec{r}}_{S}\right)}}{4\pi \left|\vec{r}-{\vec{r}}_{S}\right|}\cos\left(\theta \right)dx\;dz$$

where *k* is the wavenumber, θ is the reflection angle at each point of the measurement area, $\vec{r_{0}}$ and *r⃗*are the position vector of the emitter and the receiver respectively with respect to the coordinates system (OXY) and $\vec{r_{S}}$ is the position vector of the *dS* (defined as *dS* = *dx*·*dy*) (Figure 1b).

If the directivities of the transducers (*Q*(α) and *Q*(α')) for the emitter and receiver respectively are considered (with α the angle defined by the vector *r⃗_s_*−*r⃗*_0_ and the orientation of the emitter, and α' the angle defined by the vector *r⃗*−*r⃗_s_* and the orientation of the receiver), the Equation (1) can be rewritten as:

(2) $$p(r)=-2jk\iint_{S}\frac{e^{-j\vec{k}\left({\vec{r}}_{s}-{\vec{r}}_{0}\right)}}{4\pi \left|{\vec{r}}_{s}-{\vec{r}}_{0}\right|}Q\left(\alpha \right)\frac{e^{-j\vec{k}\left(\vec{r}-{\vec{r}}_{S}\right)}}{4\pi \left|\vec{r}-{\vec{r}}_{S}\right|}Q\left(\alpha '\right)\cos\left(\theta \right)dx\;dz$$

If we have an accurate numerical description of the surface, we can calculate the complex scattered pressure on the rough paint by means of Equation (2) for comparison with the experimental results. Comparison of the image obtained in the theoretical stage with the experimental image obtained using the PSA method provides relevant information to validate the proposed method.

## 3. Experimental Setup and Methodology

### 3.1. Experimental Setup

**Figure 2.**
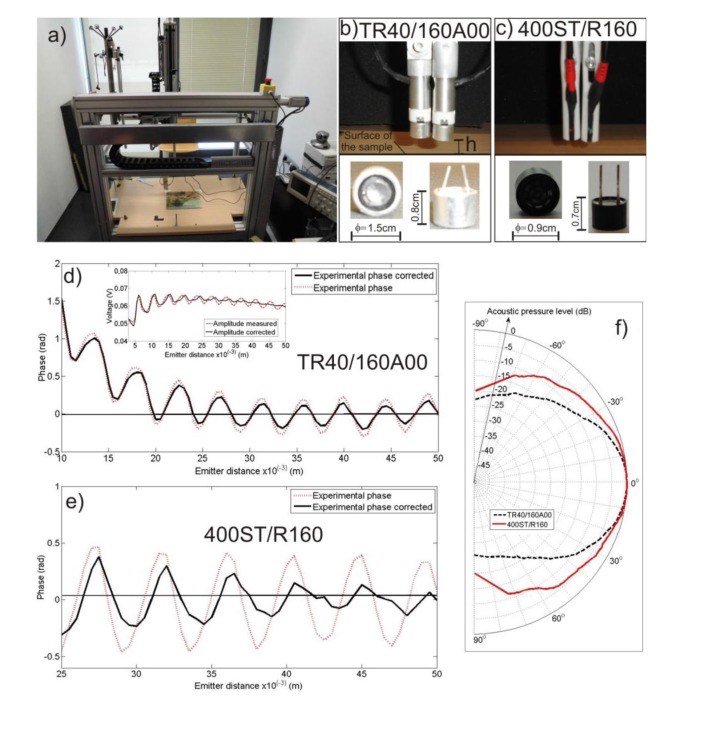
(**a**) Experimental setup: photograph of a general view of the 3D Robotized measurement system; (**b**,**c**) Arrangement of the transmitter/receiver transducers. At the bottom one can see the main geometrical characteristics of the transducers; (**d**,**e**) Experimental phase and the fit for both pairs of transducers as a function of the distance *h*. The inset in (**d**) shows the experimental phase and the fit for the corresponding transducer. (**f**) Directivity pattern measured for both transmitters.

The measurements have been carried out using an automated position system that provides a highly accurate *XYZ* movement. This measurement system is designed as a compact device to control the movement of the positioning system and, to register synchronously the measured data. Both the transmitter and the receiver transducers are moved jointly, and their movement is varied by using a Cartesian robot, which controls the movement along the three axes. The transducers move in parallel to the surface of the paint (position in semi-axis *OZ* constant, *h*, at any measure) controlling the position changes by semi-axes *OX* and *OY* inside the work area. The transmitter transducer continuously emits a sinusoidal signal of 40 kHz. When the robotized system reaches a measuring position an external trigger acts and the transducer receiver acquires the temporal signal to obtain both phase and amplitude data. To generate the signal a NI PXI-5402 14-bit card (National Instruments, Austin, TX, USA) is used, and trigger control and the obtaining of the signal is done by means of a National Instruments NI5112 Digital Oscilloscope. A photograph of the Cartesian robot used can be seen in Figure 2a.

Two pairs of emitter and receiver transducers are used in the experiments to demonstrate the robustness of the PSA technique. The arrangement of the two pairs of transducers used for measurements along with their main geometric dimension can be seen in Figure 2b,c, and Table 1 shows their technical characteristics.

### 3.2. Methodology

Careful calibration of the experimental measurement system is necessary to carry out the measures successfully. First of all, we obtained experimentally the resolution in the *OZ* movement of our experimental setup by means of the relationship between this *OZ* movement and the phase information. It is known that this resolution depends on phase sensitivity [20].

The transducers are located in their measuring position (see Figure 2b,c), with axes arranged perpendicularly to a thick flat wooden surface. Distance *h* between the transducers and the flat surface is varied in a range between 10·10^−3^ m and 50·10^−3^ m, measuring at steps of 5·10^−4^ m, the minimum step in the movement of the robot. The distance between centers of transducers is constant and equal to 17·10^−3^ m throughout the experiment.

The experimental results for the measured and unwrapped phase show a pattern characterised by a strong linear dependence between phase and distance, with an additional oscillating term, far away from the linear relationship corresponding to ideal conditions. Figure 2d,e shows these patterns for both pairs of transducers once the linear term has been subtracted. Analyzing the pattern of the oscillating term presented in Figure 2d and taking into account the amplitude pattern obtained in the experiment for the TR40 transducer (see inset in Figure 2d), one can observe that a portion of the direct field emission is captured by the receiver and causes interferences with the reflected wave, providing the characteristic crests and troughs of both patterns.

Furthermore, when transducers are near to the flat surface, a stationary wave appears that makes it difficult to characterise the expected linear regression. Therefore, we have established a working distance between 40·10^−3^ m and 50·10^−3^ m, outside the region where the stationary wave is presented. When transducers are placed around this measurement distance, the phase error due to interference phenomena takes reasonable values, a maximum of 0.25 rad for the directive transducer TR40 (Figure 2d), and 0.45 rad for the 400 ST one (Figure 2e).

From the slope of the linear regressions it is possible to calculate for both pairs of transducers the real wavelength at working conditions, λ(TR40) = 8.68·10^−3^ m, λ(400ST) = 8.75·10^−3^ m, and the ratio phase/distance resolution, ratio(TR40) = 82,910 deg/m and ratio(400ST) = 82,270 deg/m. The obtained ratios directly relate the measured phase to the distance between the pair of transducers and the reflective surface. The calculated experimental error is less than 0.8%.

Our second analysis relates to the direct contribution of the emitter to the receiver due to the proximity of both transducers in the experimental setup, so that part of the energy used to irradiate the artwork is directly measured by the receiver. This contribution is relevant for the analysis and it should be detected. To do that, we placed both pairs of transducers in an anechoic chamber with the measuring arrangement shown in Figure 2b,c but without the flat surface. Then, we excited the transducers at their working frequency with controlled amplitude of 1 V. In these conditions, the only contribution at the receiver comes directly from the emitter. The direct field amplitude registered by the receivers, *A_d_*, directly in volts is *A_d_*(TR40) = 5.15·10^−3^ V and *A_d_*(400ST) = 32.5·10^−3^ V.

In order to obtain the reflected signal without the direct contribution, the complex amplitude of the reflected wave *A̅_r_* is obtained in module and phase form as:

(3) $${\bar{A}}_{r}=\left(A_{m}\cos\beta -A_{d}\right)+j\left(A_{m}\sin\beta \right)$$

where *A_m_* and *A_d_* are respectively the amplitude in volts of the measured signal and of the direct field, and β is the relative phase of the measured signal, triggered with the direct field signal.

Finally, we characterize experimentally the directivity pattern (Q(α)) of both pairs of transducers. Figure 2f shows the directivity function in dB for the working frequency, *f* = 40 kHz, and for a distance chosen for measurements. To obtain the directivity pattern, measurements are taken at each degree by rotating the emitter from −90° to 90°.

The range of variation in height of the roughness on the artwork surface is limited by the extreme values of the phase shift φ, which is between 0° and 360°. However, this is not a limitation when the maximum depth of the artwork is smaller than half the wavelength (*f* = 40 kHz that corresponds to a λ = 85·10^−3^ m), as in our case. Similarly, if the artwork only has slow changes in its depth, this limitation can be overcome by unwrapping the phase registered for the test specimen as well.

## 4. Results and Discussion

A novel application of the PSA method that provides a useful integrated encoding image for authenticating paintings on rigid support is proposed. As already explained, the PSA technique is based on analysis of the phase shift of an ultrasonic signal after it impinges on the rough surface of the paint. Phase shift is affected mainly by: (i) scattering produced when the signal impinges on the rough surface and (ii) reflection of a part of the transmitted signal due to changes in elastic properties inside the painted wooden panel. The resulting interference pattern is formed as the sum of these two effects and is recorded in the signal captured by the receiver.

**Figure 3.**
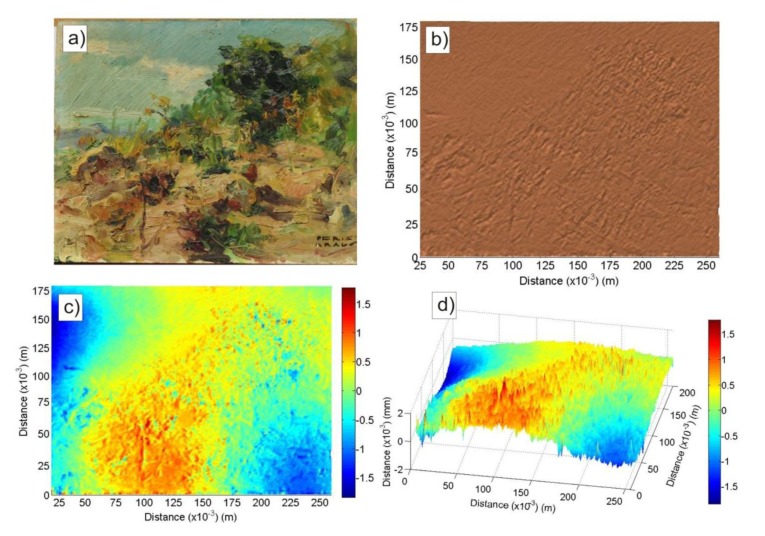
(**a**) Photograph of the panel painting used to check the PSA method presented in this work; (**b**–**d**) Photogrammetric scanning image results: (**b**) monochrome two-dimensional view; (**c**) two-dimensional and (**d**) three-dimensional views with colour scale where the relative height of the brushstrokes is represented.

To check the validity of our technique, an accurate topography of the surface roughness of Peris Aragó wooden panel painting has been done using a scanning technique based on photogrammetric correlation. Figure 3 shows the optical method results.

Figure 3a,b shows Peris Aragó's artwork and a two-dimensional monochrome view of the topography obtained by means of optical scanning respectively. Figure 3c,d both present two and three-dimensional views of the optical topography. The detailed relief formed by the brush strokes on the wooden base can be seen. This scanning represents an accurate topography of the artwork. The obtained image (surface S) gives us reliable information about the surface of the artwork and can help us to check the validation of the proposed PSA technique.

Based on the topographic information data from the surface S, the next step was to calculate the theoretical phase shift of the scattered field over the surface of the paint using Equation (2). The calculation has been performed with the same distance intervals used in the experimental process (3·10^−3^ m). Thus, both theoretical and experimental images are obtained at the same resolution and the results can be compared.

**Figure 4.**
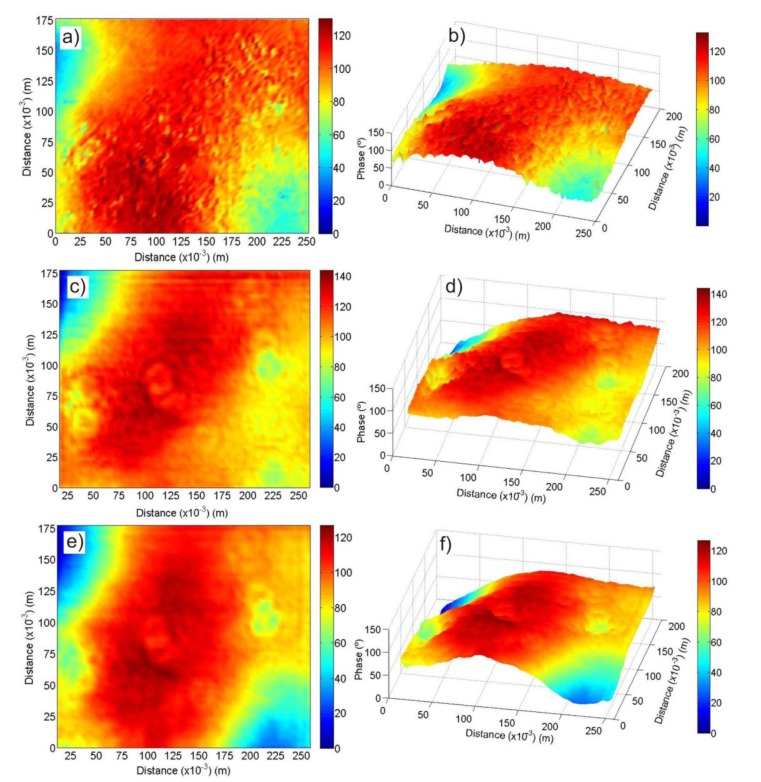
(**a**,**b**) Fresnel image results; (**c**,**d**) PSA image results for the TR40 transducers; (**e**,**f**) PSA image results for the 400ST transducers. A two-dimensional and three-dimensional view of each section is presented.

Considering the direct contribution of the emitter to the receiver due to the proximity of both transducers, we have obtained a simulation that represents the scattered field plus the direct one. Results of this simulation, called Fresnel image by us, are represented in Figure 4a,b respectively. Figure 4c–f represents two and three-dimensional views of the PSA images obtained using both the TR40 and 400ST pairs of transducers respectively.

Comparison of the theoretical Fresnel image (Figure 4a,b) and the experimental results obtained with the PSA method (Figure 4c–f) provide important information. As both outcomes are not equal, the experimental results not only give us information about the scattered field on the surface but also give information about the second phenomenon involved: the reflection of part of the transmitted wave reflected inside the paint by changes in the elastic properties of the wooden paint.

Actually this is the key feature of the present work: by scanning the artwork using PSA technique, the combined effect of the scattered acoustic pressure on the rough surface together with some internal features of the panel painting provides a “fingerprint” of the artwork.

That is, if the painting could be copied with perfect imitation of the author's style, the same composition of pigments, and even with the help of technology you could reproduce the topography of the painting, but it would not be possible to reproduce the coding obtained by the PSA method.

As explained above, we have measured with two different setups, involving different transducers and measurement heights, to demonstrate the robustness of our method. In fact, observation of the PSA images shows no evident differences between the images obtained by both setups, retaining the main features despite the change in setup configuration and directivity of the sensors.

Another phenomenon to take into account is ultrasound speckles. This phenomenon appears through interference from the scattered field. However, the goal of this work is to note the underlying phenomena in the PSA image and, therefore, analysis of the ultrasound speckle is necessary to confirm that our analysis goes further than a paint surface analysis study.

More detailed studies are needed to demonstrate the reflection of part of the transmitted wave by the inside of the artwork and, more importantly, to quantify its value and its contribution to the final PSA ultrasound image. These issues are the major challenge guiding our future work.

## 5. Conclusions

Our study provides a new application of the PSA method to obtain integrated encoding images of a wooden panel painting but it can be extrapolated to paintings on any rigid support, and can be catalogued as NDT using air as the medium of impedance adaptation.

Analyzing the results, we can conclude that the PSA method not only provides information on the relief of the sample surface but also provides a real fingerprint of the artwork due to ultrasound penetration inside it.

To verify the PSA method results, a theoretical map of phase shift was calculated, based on high-precision topographic information.

Also, the robustness of the PSA method has been demonstrated by using two pairs of transducers with different characteristics, obtaining similar results.

Cataloging and protecting artworks can be enriched with the proposed PSA technique, and the robustness of the method and the use of low cost sensors make this technique especially interesting.

## Figures and Tables

**Table 1.** Main technical characteristics of the transducers used.

**Transducers**	**TR40-16OA00**	**400ST/R160**
Centre Frequency (kHz)	40.0 ± 1.0	40.0 ± 1.0
Sound pressure level (dB)	≥115	≥120
Sensitivity (dB)	≥−65	≥−65
Capacitance (pF)	2100 ± 20%	2400 ± 20%
Max. Driving Voltage (*V*_rms_)	20	20
